# Protein Tyrosine Phosphatase Inhibition Prevents Experimental Cerebral Malaria by Precluding CXCR3 Expression on T Cells

**DOI:** 10.1038/s41598-017-05609-1

**Published:** 2017-07-14

**Authors:** Kristin M. Van Den Ham, Logan K. Smith, Martin J. Richer, Martin Olivier

**Affiliations:** 1 Department of Microbiology and Immunology, McGill University, Montréal, QC H3A 0G4 Canada; 2 Microbiome and Disease Tolerance Centre and Associate Member, Goodman Cancer Research Centre, McGill University, Montréal, QC H3A 2B4 Canada; 3 Infectious Diseases and Immunity in Global Health Program, Research Institute of the McGill University Health Centre, Montréal, QC H4A 3J1 Canada

## Abstract

Cerebral malaria induced by *Plasmodium berghei* ANKA infection is dependent on the sequestration of cytotoxic T cells within the brain and augmentation of the inflammatory response. Herein, we demonstrate that inhibition of protein tyrosine phosphatase (PTP) activity significantly attenuates T cell sequestration within the brain and prevents the development of neuropathology. Mechanistically, the initial upregulation of CXCR3 on splenic T cells upon T cell receptor stimulation was critically decreased through the reduction of T cell-intrinsic PTP activity. Furthermore, PTP inhibition markedly increased IL-10 production by splenic CD4^+^ T cells by enhancing the frequency of LAG3^+^CD49b^+^ type 1 regulatory cells. Overall, these findings demonstrate that modulation of PTP activity could possibly be utilized in the treatment of cerebral malaria and other CXCR3-mediated diseases.

## Introduction

Nearly half of the world’s population is at risk of malaria, a mosquito-borne, infectious disease caused by *Plasmodium* parasites. Notably, infection with *P. falciparum* can cause severe complications that often result in death^1^. Multiple mouse models have been employed to recapitulate and characterize the varying pathologies. Infection with *Plasmodium berghei* NK65 induces immune-mediated liver damage^2^, while infection with *P. berghei* ANKA results in a neuropathology referred to as experimental cerebral malaria (ECM)^3^. Additionally, liver damage has also been reported in this model^4, 5^.

Sequestration of cytotoxic CD8^+^ T cells within the brain is required for the disruption of the blood-brain barrier and the development of cerebral damage during *P. berghei* ANKA infection^3, 6^. The CD8^+^ T cell response is primed in the spleen^7^ through the cross-presentation of antigen by dendritic cells^8^, and the resulting upregulation of the chemokine receptor CXCR3 is necessary for the chemotaxis of T cells to the brain^9–12^. Furthermore, while a potent inflammatory response is required to control parasitemia and resolve the infection, inappropriate regulation of cytokine production can promote fatal hepatic and cerebral pathology.

The role of inflammation in ECM is poorly defined. IL-10 is an important immune regulator that can suppress inflammation^13^. Depletion of IL-10 in resistant BALB/c mice was shown to increase the incidence of ECM, and exogenous IL-10 decreased neuropathology in susceptible CBA/J mice^14^. However, in C57BL/6 mice, depletion of the IL-10 receptor did not affect susceptibility to ECM, but did significantly increase parasite burden^7^. Furthermore, IL-10 production by Foxp3^−^ regulatory CD4^+^ T cells has been shown to mitigate pathology in non-cerebral murine malaria^15, 16^. Type 1 regulatory (Tr1) cells suppress effector T cell responses through the production of high levels of IL-10^17^, and the surface markers CD49b and lymphocyte activation gene 3 (LAG-3) were recently shown to be able to non-ambiguously identify Tr1 cells^18^.

Trafficking of T cells to the brain has been established to be absolutely critical in the development of ECM^9–12^. Induction of CXCR3 requires transient T cell receptor (TCR) stimulation^19^; however the subsequent pathways that control its expression are unclear. Signal transduction downstream of TCR stimulation relies on a dynamic tyrosine phosphorylation cascade, regulated by the opposing activities of protein tyrosine kinases (PTKs) and protein tyrosine phosphatases (PTPs)^20^. For example, the PTP CD45 is crucially involved in promoting proximal TCR signalling by dephosphorylating the inhibitory tyrosine of Lck (Y505)^20^. Inhibition of PTP activity has been shown to cause at least partial T cell activation^21, 22^, but the impact of PTP inhibition in conjunction with TCR stimulation is unknown.

PTP activity is regulated by a variety of physiological mechanisms, including dimerization^23^, oxidation^24^ and increased systemic levels of iron^25^. Furthermore, PTP inhibition has been shown to reduce pathology in models of asthma^26^, cancer^27^ and leishmaniasis^28^. However, the underlying pathological mechanisms that are modulated by tyrosine phosphorylation are largely undefined, thus we were interested in examining the impact of direct PTP inhibition on the T cell response and on the regulation of infection-induced inflammation during ECM.

We determined that treatment with the PTP inhibitor potassium bisperoxo (1, 10-phenanthroline) oxovanadate (V) trihydrate (bpV(phen)), precluded the development of hepatic and cerebral damage in ECM. PTP inhibition significantly decreased the brain sequestration of CD4^+^ and CD8^+^ T cells, concomitant with a marked decrease in the expression of CXCR3 on splenic T cells. bpV(phen) prevented the initial upregulation of CXCR3, which was associated with differential tyrosine phosphorylation of the proximal TCR-signalling molecule Lck. Moreover, PTP inhibition greatly augmented the frequency of IL-10-producing regulatory CD4^+^ T cells, and both bpV(phen) and IL-10 were shown to limit hepatic pathology. Thus, we have demonstrated that modulation of PTP activity has the potential to be utilized in the development of novel adjunctive therapies for malaria.

## Results

### Inhibition of PTP activity prevents the development of ECM

**Figure 1:**
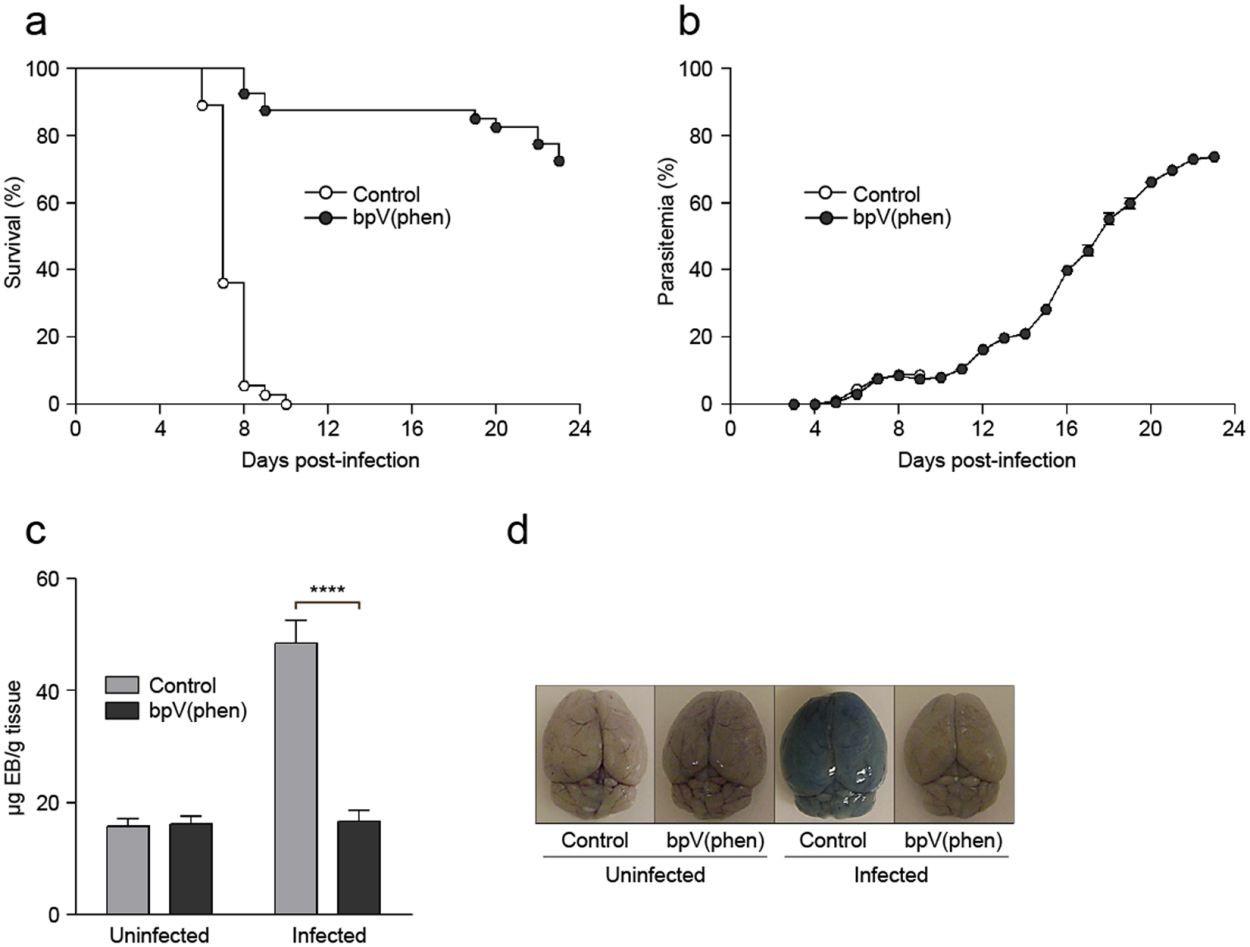
PTP inhibition prevents the development of ECM. (**a**) Survival and (**b**) parasitemia of bpV(phen)-treated mice infected with *P. berghei* ANKA, (**c**) graph of Evans blue accumulation within the brain and (**d**) representative picture of Evans blue-stained brains. For survival and parasitemia, the cumulative average of six independent experiments is shown. *n* = 36 for control mice and *n* = 40 for bpV(phen)-treated mice. P < 0.0001 using the log-rank test. Evans blue assay was performed on day 7 post-infection. *n* = 5 for uninfected, control mice, *n* = 4 for uninfected, bpV(phen)-treated mice, *n* = 7 for infected, control mice and *n* = 5 for infected, bpV(phen)-treated mice for the Evans blue assay. ****P < 0.0001 using a one-way ANOVA and Tukey’s multiple comparisons test.

To determine the impact of reduced tyrosine phosphatase activity on the pathology of ECM, mice were treated with the PTP inhibitor, bpV(phen), daily from 3 days before to 12 days after infection with *P. berghei* ANKA. bpV(phen) targets a conserved catalytic cysteine, resulting in a general inhibition of PTP activity^29, 30^. While 100% of the control mice succumbed to ECM, the bpV(phen)-treated mice were markedly protected, with an overall ECM incidence of less than 13% (Fig. 1a). Furthermore, the parasitemia of the control and bpV(phen)-treated mice was similar until the control mice succumbed to the infection, indicating that the protective effect of PTP inhibition did not rely on the increased clearance of parasites (Fig. 1b). The bpV(phen)-treated mice that did not develop ECM had increasing levels of parasitemia and either succumbed to hyperparasitemia or were sacrificed on day 23 post-infection. The incidence of ECM was confirmed by examining the integrity of the blood-brain barrier using Evans blue. The uptake of the dye into the brain parenchyma, indicative of blood-brain barrier disruption, was significantly decreased in the infected bpV(phen)-treated mice (Fig. 1c and d). Moreover, the control mice developed definite symptoms of neuropathology, but PTP inhibition prevented the clinical manifestation of cerebral malaria (see Supplementary Fig. S1). Therefore, inhibition of PTP activity is capable of protecting mice from ECM by preventing the development of neuropathology.

### bpV(phen) treatment enhances IL-10 production by splenic regulatory CD4^+^ T cells

PTP inhibition using bpV(phen) has been shown to modulate the inflammatory response during leishmaniasis, asthma and cancer^26, 27, 31^, and previously IL-10 was shown to afford protection against ECM^14^. The effect of Tregs on the cerebral pathology induced by *P. berghei* ANKA infection is still uncertain^32–36^, but IL-10 production by Foxp3^−^CD4^+^ T cells has been reported to decrease pathology in non-cerebral murine malaria^15, 16^. Thus, we asked whether PTP inhibition protected mice from ECM by modulating immunoregulatory mechanisms.

**Figure 2:**
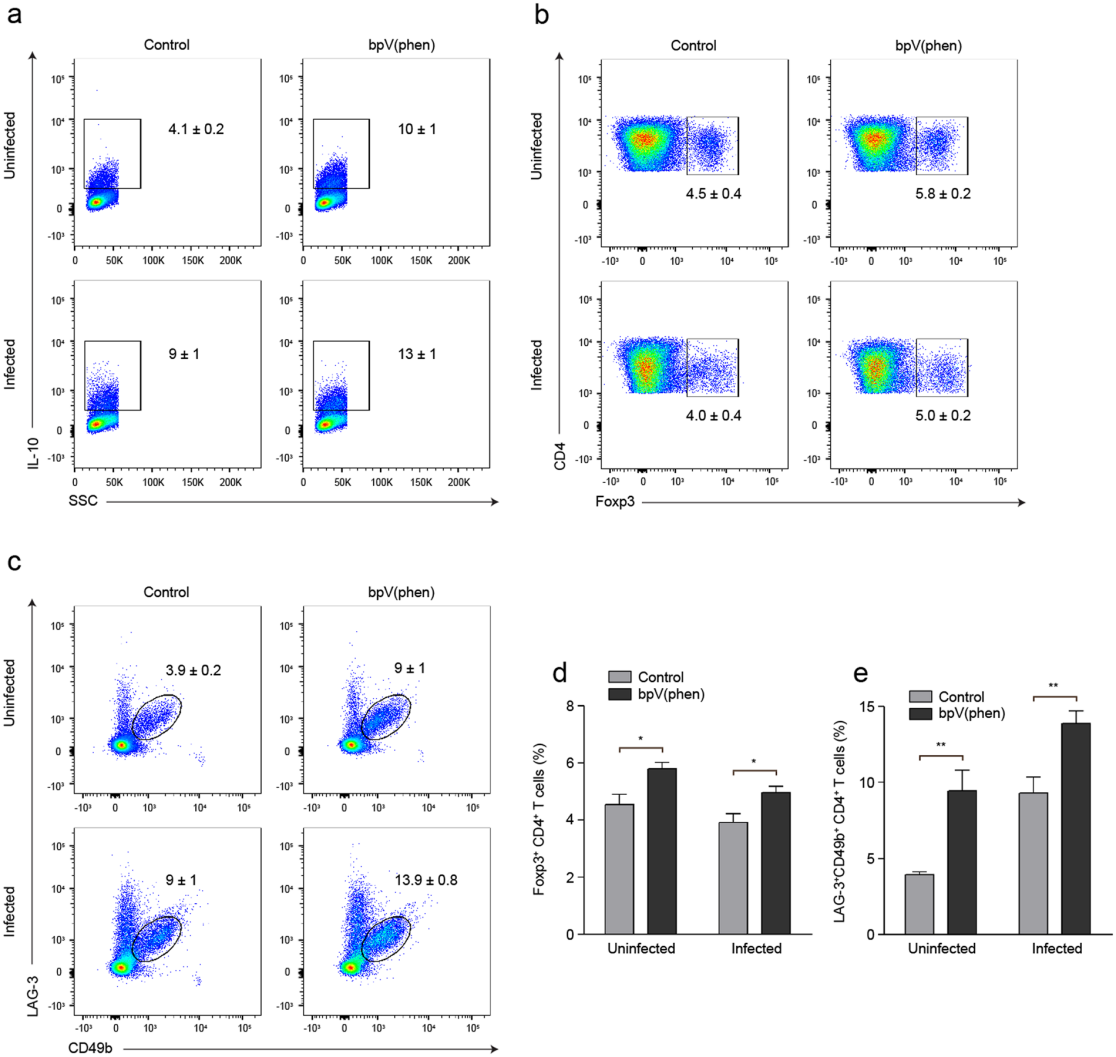
bpV(phen) treatment increases the frequency of regulatory CD4^+^ T cells in the spleen. Representative flow cytometry plots of (**a**) IL-10^+^ CD4^+^ T cells, (**b**) Foxp3^+^ and (**c**) LAG-3^+^CD49b^+^ CD4^+^ T cells, and the percentage of (**d**) Foxp3^+^ and (**e**) LAG-3^+^CD49b^+^ CD4^+^ T cells measured on day 7 post-infection after PMA/ionomycin stimulation. The numbers shown on the flow cytometry plots indicate the mean percentage of cells inside the gate ± S.E.M. The cumulative average of 2 independent experiments is shown. *n* = 9 for uninfected, control mice, *n* = 9 for uninfected, bpV(phen)-treated mice, *n* = 11 for infected, control mice, and *n* = 11 for infected, bpV(phen)-treated mice. For the IL-10^+^CD4^+^ T cells, P = 0.0047 for the uninfected control and bpV(phen)-treated mice, and P = 0.0263 of the infected control and bpV(phen)-treated mice. *P < 0.05 and **P < 0.01. Statistical significance was determined using a one-way ANOVA and Tukey’s multiple comparisons test.

The percentage of IL-10^+^CD4^+^ T cells in the spleen increased with both infection and bpV(phen) treatment (Fig. 2a). PTP inhibition caused a slight increase in the percentage of Foxp3^+^ CD4^+^ T cells in both the uninfected and infected mice (Fig. 2b and d). However, Tregs only accounted for approximately 10% of the total IL-10^+^CD4^+^ T cells in the uninfected mice and slightly more than 15% of the total IL-10^+^CD4^+^ T cells in the infected mice, congruous with an infection-dependent increase in IL-10 production by Foxp3^+^CD4^+^ T cells (see Supplementary Fig. S2). Thus, despite being positively regulated by PTP inhibition, Foxp3^+^CD4^+^ T cells only represented a small fraction of the enhanced IL-10 production induced by bpV(phen) treatment.

Next, we examined the impact of PTP inhibition on splenic Tr1 cells. The frequency of CD4^+^ T cells expressing both LAG3 and CD49b (Tr1 cells) increased with infection and was further enhanced by bpV(phen) treatment (Fig. 2c and e). Importantly, Tr1 cells accounted for the majority of the IL-10 producing CD4^+^ T cells; greater than 75% of the IL-10^+^CD4^+^ T cells also expressed LAG3 and CD49b (see Supplementary Fig. S2). Thus, the bpV(phen)-mediated increase in IL-10 production by splenic CD4^+^ T cells is likely the result of the concomitant increase in the frequency of LAG3^+^CD49b^+^ Tr1 cells.

IFNγ/IL-10 co-producing cells were shown to be the principal source of IL-10 during *P. chabaudi* infection^15^, and adoptive transfer of IL-10 producing regulatory B cells has been shown to decrease the incidence of ECM^37^, thus we examined the frequency of double positive CD4^+^ T cells and IL-10^+^ B cells. The percentage of IFNγ^+^IL-10^+^ CD4^+^ T cells increased with infection, but PTP inhibition caused a trend toward a reduction in the frequency of double-producing CD4^+^ T cells in the infected mice, but this difference did not reach statistical significance (see Supplementary Fig. S2). Moreover, infection also increased the production of IL-10 by B cells in the spleen, but PTP inhibition had no effect on the percentage of IL-10^+^ B cells (see Supplementary Fig. S2). Taken together, this data suggests that although *P. berghei* ANKA infection increases the expression of IL-10 by other cellular sources, IL-10 production by these cell subsets is not positively regulated by PTP inhibition.

### IL-10 reduces mortality from P. berghei ANKA infection independently of cerebral pathology

**Figure 3:**
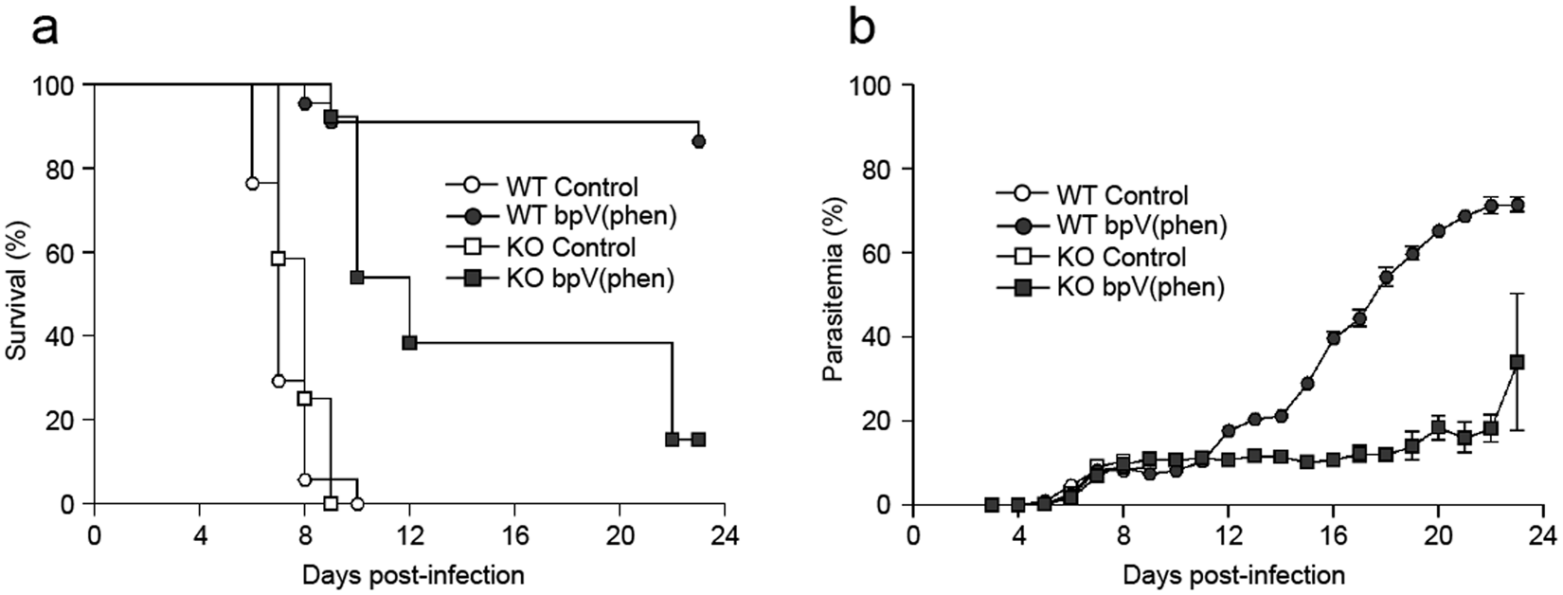
PTP inhibition partially protects IL-10KO mice from *P. berghei* ANKA infection. (**a**) Survival and (**b**) parasitemia of bpV(phen)-treated WT and IL-10KO mice infected with *P. berghei* ANKA. The cumulative average of 4 independent experiments is shown. *n* = 17 for control, WT mice, *n* = 22 for bpV(phen)-treated, WT mice, *n* = 12 for control, IL-10KO mice and *n* = 13 for bpV(phen)-treated, IL-10KO mice. For bpV(phen)-treated, WT mice versus bpV(phen)-treated IL-10KO mice, P < 0.0001, and for control, IL-10KO mice versus bpV(phen)-treated IL-10KO mice, P < 0.0001, using the log-rank test.

To determine if the bpV(phen)-mediated protection from ECM was IL-10 dependent, IL-10 knock-out (IL-10KO) mice were treated with bpV(phen) following the same protocol that was used with the wild-type (WT) mice. The onset and clinical manifestation of ECM in the untreated WT and IL-10KO mice was not significantly different, suggesting that IL-10 does not have an essential role in the development of cerebral pathology during *P. berghei* ANKA infection (Fig. 3a; see Supplementary Fig. S3).

bpV(phen)-treated IL-10KO mice were partially protected, with nearly 40% of the mice surviving until 3 weeks post-infection. The parasitemia of the bpV(phen)-treated WT and IL-10KO mice was similar until 11 days post-infection; and as the infection progressed, the surviving, bpV(phen)-treated IL-10KO mice demonstrated superior control of their blood parasite levels (Fig. 3b). Importantly, the bpV(phen)-treated IL-10KO mice that succumbed to the infection did not present with any of the typical clinical symptoms of cerebral malaria, such as paralysis or coma, but instead developed weakness, hypothermia and pallor (see Supplementary Fig. S3). The absence of cerebral symptoms in the bpV(phen)-treated IL-10KO mice demonstrates that IL-10 is not required to preclude the development of neuropathology following PTP inhibition; however, the increased percentage of IL-10KO mice succumbing to the infection at early time points indicates that IL-10 has a key role in limiting *P. berghei* ANKA-induced pathology.

### bpV(phen) and IL-10 attenuate P. berghei ANKA-induced liver injury

**Figure 4:**
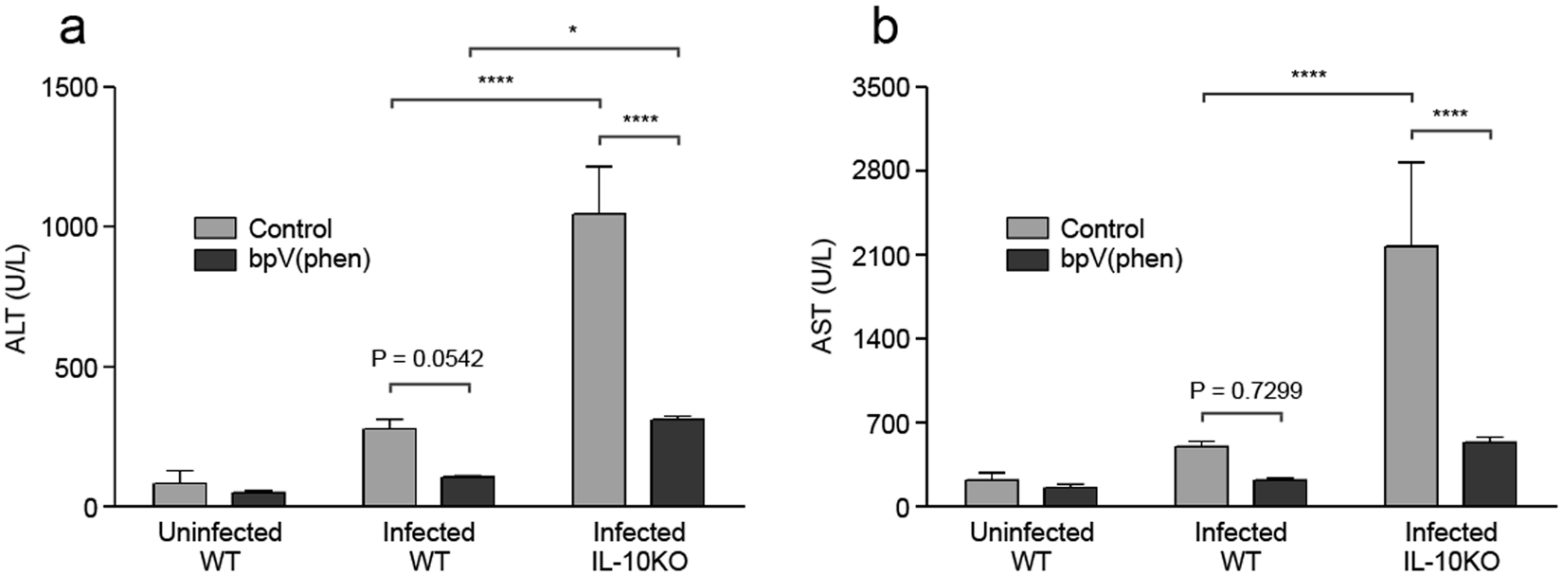
bpV(phen) treatment and IL-10 mitigate ECM-induced liver damage. Serum levels of (**a**) ALT and (**b**) AST measured on day 7 (control WT and IL-10KO mice) and day 10 (bpV(phen)-treated WT and IL-10KO mice) post-infection. *n* = 5 for control, WT mice, *n* = 8 for bpV(phen)-treated, WT mice, *n* = 4 for control, IL-10KO mice and *n* = 4 for bpV(phen)-treated, IL-10KO mice. *P < 0.05 and ****P < 0.0001 using a one-way ANOVA and Tukey’s multiple comparisons test.

In addition to cerebral damage, severe malaria can also present as hyperparasitemia-induced anemia and immune-mediated liver injury. Since the parasitemia of the bpV(phen)-treated IL-10KO mice was similar to the bpV(phen)-treated WT mice when they died (days 9 to 12 post-infection), we examined whether IL-10 was involved in controlling liver injury by measuring the serum levels of alanine transaminase (ALT) and aspartate transaminase (AST) (Fig. 4a and b). Control WT and IL-10KO mice were analyzed 7 days post-infection, upon the onset of neurological symptoms in both groups, and the bpV(phen)-treated WT and IL-10KO mice were evaluated on day 10 post-infection, when the IL-10KO mice began to develop weakness, pallor and hypothermia. PTP inhibition had no effect on the serum levels of ALT or AST in the uninfected mice. The absence of IL-10 markedly increased liver injury in both the control and bpV(phen)-treated mice, while treatment with bpV(phen) significantly decreased the serum levels of ALT and AST in the IL-10KO mice and further attenuated hepatic pathology in the WT mice.

Large parasite burdens were previously associated with liver damage during *P. berghei* ANKA infection^5^, and IL-10 has been shown to limit parasite sequestration during ECM^7^, therefore we examined liver parasite burden as a possible source of liver damage in our model. The parasite burden was measured as both RLU/mg tissue and total RLU per liver (see Supplementary Fig. S4), as the bpV(phen)-treated WT and IL-10KO mice had slightly enlarged livers compared to the control mice. Neither bpV(phen) treatment nor the presence of IL-10 affected the liver parasite burden. Moreover, the accumulation of parasites within the liver did not appear to be directly responsible for increased hepatic pathology, but rather occurred subsequent to cerebral damage. This data demonstrates that PTP inhibition and IL-10 are capable of attenuating liver injury independently of liver parasite burden.

### PTP inhibition significantly decreases the brain sequestration of CD4^+^ and CD8^+^ T cells

**Figure 5:**
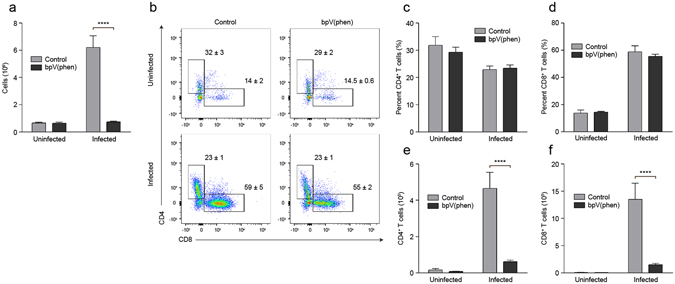
PTP inhibition prevents the brain sequestration of CD4^+^ and CD8^+^ T cells. (**a**) Total cells sequestered in the brain, (**b**) representative flow cytometry plots of CD4^+^ and CD8^+^ T cells, percentage of brain-infiltrating leukocytes that are (**c**) CD4^+^ T cells and (**d**) CD8^+^ T cells, and total brain-sequestered (**e**) CD4^+^ T cells and (**f**) CD8^+^ T cells measured on day 7 post-infection. Infiltrating leukocytes are defined as CD11b^lo-hi^CD45^+^ cells. The numbers shown on the flow cytometry plots indicate the mean percentage of cells inside the gate ± S.E.M. *n* = 5 for uninfected, control mice, *n* = 5 for uninfected, bpV(phen)-treated mice, *n* = 5 for infected, control mice and *n* = 8 for infected, bpV(phen)-treated mice. ****P < 0.0001 using a one-way ANOVA and Tukey’s multiple comparisons test.

Sequestration of cytotoxic CD8^+^ T cells within the brain microvasculature is required for the development of neuropathology during *P. berghei* ANKA infection^3, 6^. Strikingly, bpV(phen) treatment inhibited the infection-induced infiltration of cells into the brain (Fig. 5a). The percentage of sequestered cells that were CD4^+^ or CD8^+^ T cells was not affected by bpV(phen), but infection reduced the percentage of CD4^+^ T cells and increased the percentage of CD8^+^ T cells (Fig. 5b–d). Importantly the total number of CD4^+^ and CD8^+^ T cells that accumulated within the brain was markedly decreased by PTP inhibition in the infected mice (Fig. 5e and f). These results indicate that PTP inhibition prevents the development of neuropathology by preventing the sequestration of both CD4^+^ and CD8^+^ T cells within the brain.

### bpV(phen) decreases CXCR3 expression on splenic T cells

**Figure 6:**
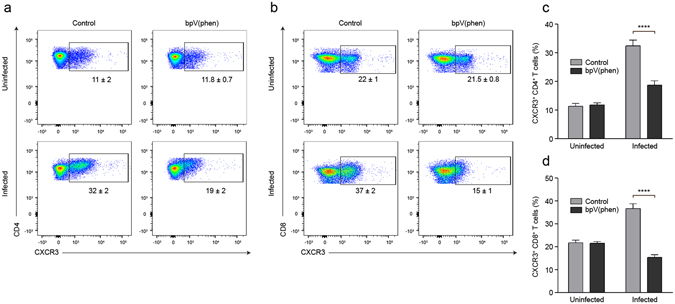
bpV(phen) treatment attenuates the expression of CXCR3 on splenic CD4^+^ and CD8^+^ T cells. Representative flow cytometry plots of CXCR3^+^ (**a**) CD4^+^ and (**b**) CD8^+^ T cells, and the percentage of CXCR3^+^ (**c**) CD4^+^ and (**d**) CD8^+^ T cells measured on day 7 post-infection. The numbers shown on the flow cytometry plots indicate the mean percentage of cells inside the gate ± S.E.M. The cumulative average of 2 independent experiments is shown. *n* = 9 for uninfected, control mice, *n* = 10 for uninfected, bpV(phen)-treated mice, *n* = 9 for infected, control mice, and *n* = 9 for infected, bpV(phen)-treated mice. ****P < 0.0001 using a one-way ANOVA and Tukey’s multiple comparisons test.

T cell priming occurs in the spleen^7^ and the chemokine receptor CXCR3 plays an integral role in T cell migration to the brain during *P. berghei* ANKA infection^9–12^. As our data highlighted a near complete reduction in cell sequestration with the brain, we analyzed the splenic CD4^+^ and CD8^+^ T cell populations to determine how PTP inhibition modulated the T cell response during *Plasmodium* infection in order to preclude T cell sequestration within the brain. While infection caused a significant increase in the frequency of CXCR3^+^ CD4^+^ and CD8^+^ T cells in the spleen of control mice, bpV(phen) treatment completely abrogated the upregulation of CXCR3 on T cells following infection (Fig. 6a–d).

PTP inhibition did not affect the activation of splenic CD4^+^ T cells, but the percentage of activated CD8^+^ T cells in the spleen was significantly decreased in the infected, bpV(phen)-treated mice (see Supplementary Fig. S5). Consequently, the expression of CXCR3 on activated T cells was examined to determine if the attenuated activation was responsible for the reduced frequency of CXCR3^+^ cells. The percentage of CD62L^lo^CD44^+^ CD4^+^ and CD8^+^ T cells that expressed CXCR3 was decreased by bpV(phen) treatment in a similar manner to what was observed for total CD4^+^ and CD8^+^ T cells (see Supplementary Fig. S6). Thus PTP inhibition is able to decrease the expression of CXCR3 on T cells on both total and activated splenic T cells, suggesting that bpV(phen) decreases the expression of this chemokine receptor independently of T cell activation.

Since PTP inhibition markedly decreased the activation of splenic CD8^+^ T cells, we were interested in determining if bpV(phen) treatment also affected the frequency or total number of antigen-experienced CD8^+^ T cells. We examined the total population of splenic CD8^+^ T cells exhibiting an antigen-experienced phenotype by measuring the percentage of CD8^+^ T cells that were CD11a^hi^CD8^lo^ at the onset of symptoms in the control mice^38^. bpV(phen) did not affect the frequency or total number of antigen-experienced CD8^+^ T cells induced by *P. berghei* ANKA infection (see Supplementary Fig. S6). Thus downregulation of CXCR3 expression on both CD4^+^ and CD8^+^ T cells, and the consequent attenuation of T cell chemotaxis to the brain, rather than decreased numbers of responding T cells, appears to be the main mechanism through which PTP inhibition prevents neuropathogenesis.

### PTP inhibition prevents CXCR3 upregulation by attenuating TCR signal transduction

**Figure 7:**
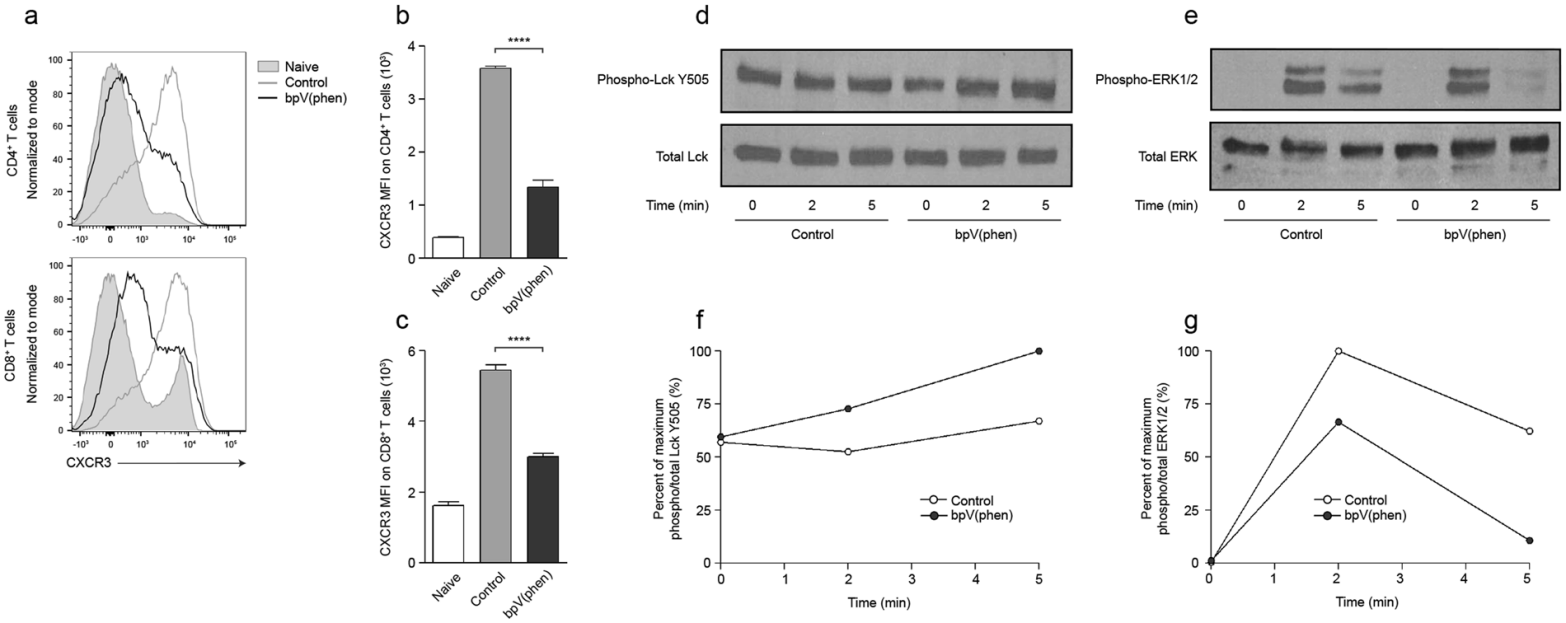
PTP inhibition prevents the upregulation of CXCR3 and attenuates TCR-mediated signalling in splenic T cells. (**a**) Representative histograms of CXCR3 expression on bpV(phen)-treated CD4^+^ and CD8^+^ T cells and the mean fluorescence intensity (MFI) of CXCR3 on bpV(phen)-treated (**b**) CD4^+^ and (**c**) CD8^+^ T cells following anti-CD3/anti-CD28 stimulation. ****P < 0.0001 using a one-way ANOVA and Tukey’s multiple comparisons test. The cumulative average of 3 independent experiments is shown. Representative western blots of (**d**) phospho- and total Lck Y505 and (**e**) phospho- and total ERK1/2, and densitometry graphs of (**f**) phospho-LckY505 and (**g**) phospho-ERK1/2. The western blots and densitometry graphs are representative of 3 independent experiments.

Since the attenuated activation of T cells was not responsible for the decreased percentage of CXCR3^+^ T cells, we were interested in identifying the mechanism used by bpV(phen) to prevent the upregulation of this chemokine receptor. Previous studies have demonstrated that CXCR3 induction requires transient TCR stimulation^19^, but the subsequent pathways that regulate CXCR3 expression are uncertain. Isolated splenic CD4^+^ and CD8^+^ T cells were stimulated with anti-CD3/anti-CD28 and then allowed to recover in the absence of stimulation to upregulate CXCR3 expression *ex vivo*. bpV(phen) significantly suppressed the induction of CXCR3 on both CD4^+^ and CD8^+^ T cells (Fig. 7a–c). CXCR3 expression was markedly reduced by PTP inhibition when bpV(phen) was present during TCR stimulation, whereas the addition of bpV(phen) during the recovery incubation did not affect the expression of CXCR3 on CD4^+^ T cells and only caused a partial decrease in CXCR3 expression on CD8^+^ T cells (see Supplementary Fig. S7).

TCR signal transduction is dependent on the concerted actions of PTKs and PTPs^20^. Lck is one of the first kinases recruited upon TCR stimulation, and the activity of this kinase is modulated by the phosphorylation of a negative regulatory tyrosine (Y505) and an opposing positive regulatory tyrosine (Y394). CD45-dependent dephosphorylation of the negative regulatory site is required for efficient TCR-mediated signalling. Following the same stimulation protocol used above, the effect of bpV(phen) on TCR signal transduction was further examined in CD8^+^ T cells. bpV(phen) treatment caused an increase in the phosphorylation of the negative regulatory tyrosine of Lck following CD3 cross-linking (Fig. 7d and f). This resulted in a decrease in the phosphorylation of the downstream kinases ERK1/2, with phospho-ERK1/2 being almost entirely lost after 5 minutes of stimulation (Fig. 7e and g). Thus, PTP inhibition attenuates the TCR signalling capacity of T cells by preventing the dephosphorylation of the Y505 residue on Lck, and this decreased signalling is likely responsible for the reduced expression of CXCR3 on T cells.

## Discussion

The development of ECM requires the accumulation of cytotoxic CD8^+^ T cells within the brain and dysregulation of the proinflammatory response. bpV(phen) has been shown to modulate the inflammatory response during *Leishmania* infection^28, 31^, but the impact of PTP inhibition on the T cell response has not previously been investigated. Herein, we demonstrate that bpV(phen) treatment throughout the infection precludes the development of cerebral and hepatic pathology during *P. berghei* ANKA infection and rescues infected animals from ECM-induced mortality. PTP inhibition attenuated cerebral damage by preventing the TCR-dependent upregulation of CXCR3 expression on splenic T cells and reduced liver injury using a mechanism that was independent of liver parasite sequestration.

PTP inhibition prevented the disruption of the blood-brain barrier and the consequent development of neuropathology by markedly decreasing the sequestration of both CD4^+^ and CD8^+^ T cells within the brain. The chemokine receptor CXCR3 has been shown to play an integral role in the trafficking of pathogenic T cells to the brain during *P. berghei* ANKA infection^9–12^. We observed that bpV(phen) treatment significantly reduced the expression of CXCR3 on CD4^+^ and CD8^+^ T cells in the spleen. PTP inhibition attenuated the activation of splenic CD8^+^ T cells, but had no impact on the activation of CD4^+^ T cells, but the percentage of activated T cells that expressed CXCR3 was also reduced by bpV(phen), indicating that PTP inhibition is sufficient to decrease CXCR3 even on activated T cells and that the decreased expression of CXCR3 was not resultant of attenuated T cell activation.

The mechanism used by PTP inhibition to reduce CXCR3 expression was further examined by analyzing the effect of bpV(phen) on isolated splenic T cells. The presence of bpV(phen) during TCR stimulation precluded the initial upregulation of CXCR3 on both CD4^+^ and CD8^+^ T cells. PTP inhibition during the recovery period following stimulation did not affect the expression of CXCR3 on CD4^+^ T cells and had only an intermediate effect on CXCR3 expression on CD8^+^ T cells. These results demonstrate that the underlying mechanisms controlling CXCR3 expression on CD4^+^ and CD8^+^ T cells are differentially regulated by tyrosine phosphorylation. For both cell types, PTP inhibition had the largest impact on CXCR3 expression during TCR stimulation, indicating that bpV(phen) predominately decreases CXCR3 expression by inhibiting a PTP involved in TCR signal transduction. Notably, PTP inhibition during the recovery phase partially decreased CXCR3 expression on CD8^+^ T cells, but the reduction was not synergistic with the decrease caused by bpV(phen) during TCR stimulation. Thus, it appears that CD8^+^ T cells have redundant, PTP-mediated mechanisms that control CXCR3 expression.

Based on the finding that PTP inhibition had the greatest effect on CXCR3 expression during TCR stimulation, the impact of bpV(phen) on TCR signal transduction was further examined. PTP inhibition increased the phosphorylation of the negative regulatory tyrosine of Lck following TCR triggering. The effect of the increased phosphorylation of Lck Y505 on TCR signalling was analyzed by measuring the phosphorylation of the downstream ERK pathway. The phosphorylation of the kinases ERK1/2 were reduced by bpV(phen), with phosphorylation being almost entirely lost 5 minutes after stimulation. Based on these results, bpV(phen) treatment is likely attenuating the TCR signalling capacity of T cells by preventing the dephosphorylation of Lck Y505 and the subsequent activation of this kinase. Dephosphorylation of the negative regulatory site of Lck is controlled by the tyrosine phosphatase CD45^20^, thus bpV(phen) is presumably precluding the expression of CXCR3 on splenic CD4^+^ and CD8^+^ T cells by inhibiting the activity of this PTP. Nevertheless, bpV(phen) causes a general inhibition of PTP activity, thus further studies will be required to determine whether other PTPs are involved in the pathogenesis of ECM.

In addition to preventing the development of neuropathology, we demonstrated that bpV(phen) is also able to modulate the inflammatory response. CD4^+^ T cells have previously been shown to be the dominant source of IL-10 during both *P. chabaudi* and *P. yoelii* infection^15, 16^. We found that the frequency of IL-10-producing CD4^+^ T cells was increased by both infection and PTP inhibition. Similar to the non-cerebral malaria infections, the majority of the IL-10^+^CD4^+^ T cells were Foxp3^−^ Tr1 cells. However, the criteria used to designate the regulatory CD4^+^ T cells as Tr1 cells differed between our studies, as the previous work was published before the identification of LAG-3 and CD49b as selective surface markers^18^. Following the determination of Tr1 cells as the dominant source of IL-10 induced by bpV(phen) treatment during *P. berghei* ANKA infection, we examined whether IL-10 contributed to the protection afforded by PTP inhibition.

Depletion of the IL-10 receptor^7^ and IL-10 deficiency^32^ in C57BL/6 mice were previously shown to have little to no effect on the incidence of ECM, and we observed that the control IL-10KO mice were not significantly protected compared to the control WT mice. PTP inhibition prevented the development of neuropathology in the IL-10KO mice, evidenced by the lack of cerebral malaria symptoms exhibited by these mice, indicating that bpV(phen)-mediated protection from ECM is not IL-10 dependent. Notably, the bpV(phen)-treated IL-10KO mice that survived past 2 weeks post-infection displayed far superior control of their parasitemia compared to the bpV(phen)-treated WT mice. IL-10KO mice have also been shown to have decreased parasitemia compared to WT mice in *P. yoelii* and *P. berghei* NK65 infection^16, 39^. The decreased parasitemia in these studies correlated with an increased serum concentration of IFNγ^16^. IFNγ is critically involved in the induction of ECM^40^; thus the prevention of neuropathology by bpV(phen) treatment may have bypassed the IFNγ-mediated pathology of this disease and allowed the increased IFNγ production of the IL-10KO mice to better control their parasitemia.

Approximately two-thirds of the bpV(phen)-treated IL-10KO succumbed to the infection nearly two weeks before the majority of the bpV(phen)-treated WT mice. Since the parasitemia of the IL-10KO and WT mice was similar during this period of time, the impact of both bpV(phen) and IL-10 on hepatic pathology was analyzed. Both PTP inhibition and the presence of IL-10 were found to reduce liver injury. A previous study associated liver damage with a high liver parasite burden^5^. Half of our control WT mice were symptomatic on day 7 post-infection, and the onset of neurological symptoms markedly increased the accumulation of parasites within the liver, but had no effect on hepatic pathology. Furthermore the non-symptomatic, control WT mice and the bpV(phen)-treated WT mice had a similar parasite burden, even though the non-symptomatic, control WT mice had increased liver damage. Thus, our results indicate that liver parasite burden is not directly responsible for hepatic pathology.

Furthermore, depletion of the IL-10 receptor was previously linked to increased tissue parasite burden^7^, suggesting that IL-10 may limit tissue sequestration. In our study, the presence of IL-10 decreased liver damage, but did not appear to have an effect on the liver parasite burden. There was a slight, but not significant, increase in parasite accumulation in the bpV(phen)-treated IL-10KO mice compared to the bpV(phen)-treated WT mice, but this is likely not caused by the lack of IL-10 production. This assumption is supported by the control mice, as there was no difference in the liver parasite burden between the symptomatic, control WT mice and the control IL-10KO (all of which were symptomatic), despite the difference in IL-10 expression. Therefore our results do not indicate that the presence of IL-10 decreases the sequestration of parasites in the liver. Furthermore, the liver parasite burden in control WT mice increased significantly upon the onset of symptoms, suggesting that parasite sequestration within this organ is dependent on the onset of cerebral pathology.

While ECM and human cerebral malaria share similar pathological mechanisms, the role of CD8^+^ T cells in the human disease remains indeterminate. A pathological role for CD8^+^ T cells has often been disregarded based on their paucity in human cerebral malaria^41^; however, intravascular CD8^+^ T cells are also difficult to identify in murine brains^3, 42^. Moreover, resistance to severe malaria in humans has been linked to certain human leukocyte antigen class I alleles^43^ and the expression of CXCL10, a chemokine for CXCR3, has been linked to disease severity^44, 45^. Thus, the data support the possibility that CD8^+^ T cells may contribute to pathology in human cerebral malaria and warrant further investigation.

Overall, our study supports earlier work which demonstrated that hepatic pathology also occurs in the *P. berghei* ANKA model and that liver injury is independent of cerebral pathology. However, we found that liver damage did not correlate with a high parasite burden and that the presence of IL-10 did not limit liver parasite burden. Moreover, our results also support the crucial role of CXCR3 and the consequent brain sequestration of T cells in the development of neuropathology. The factors influencing the expression of CXCR3 remain poorly defined, particularly during infection. We determined that T cell-intrinsic PTP(s) are critically involved in the initial upregulation of CXCR3 on T cells in ECM, and that PTP inhibition was sufficient to prevent the sequestration of pathogenic T cells within the brain. Recently, increased systemic iron levels were also shown to prevent the development of ECM by modulating the chemotactic T cell response^46^. Elevated iron levels decreased CXCR3 expression only on CD4^+^ T cells by interfering with IFNγ signalling^46^, in contrast to PTP inhibition, which reduced CXCR3 expression on both CD4^+^ and CD8^+^ T cells, likely through the modulation of TCR signal transduction.

In conclusion, we have established that regulation of tyrosine phosphorylation plays an essential role in both hepatic and cerebral pathology during *P. berghei* ANKA infection and that an improved understanding of PTP regulation and that further characterization of the specific PTPs that contribute to the pathogenesis of ECM may be beneficial to the design of novel immunotherapies for malaria. Moreover, a better understanding of the factors influencing CXCR3 expression would likely also be advantageous for other CXCR3-mediated diseases, such as graft-versus-host disease, rheumatoid arthritis and multiple sclerosis^47–52^.

## Methods

### Mice

C57BL/6 mice (6–8 weeks old) were purchased from Charles River Laboratories and IL-10KO mice on a C57BL/6 background (6–8 weeks) were purchased from The Jackson Laboratory. All mice were maintained under specific pathogen-free conditions. All research involving mice was carried out according to the regulations of the Canadian Council of Animal Care and was approved by the McGill University Animal Care Committee under ethics protocol number 5925 and the Research Institute of the McGill University Health Centre Animal Care Committee under ethics protocol number 7607. Mice were euthanized at established humane endpoints using CO_2_ asphyxiation followed by cervical dislocation or by using isoflurane if perfusion was performed.

### Parasites and infection

In all experiments, red blood cells infected with *P. berghei* ANKA parasites expressing a green fluorescent protein (GFP)-luciferase fusion protein were used (Malaria Research and Reference Reagent Resource Center). WT and IL-10KO C57BL/6 mice were infected by intraperitoneal (i.p.) inoculation of 10^4^ infected red blood cells. In all experiments mice received either PBS or 1.5µmol/18 g body weight of bpV(phen)^28^ daily from 3 days before to 12 days after parasite inoculation (infected groups) or mock infection (uninfected groups), or up until the experimental endpoint (day 7 or 10 post-infection), by subcutaneous injection. Starting on day 3 post-infection of all survival experiments, tail-vein blood was collected daily. Blood smears were stained with Diff-Quik, and parasitemia was determined by counting at least 500 cells.

### Blood-brain barrier integrity analysis

Mice were i.p. injected with 0.3 mL of 2% Evans blue (Sigma-Aldrich). The mice were sacrificed 2 h thereafter, without perfusion, and brains were weighed and placed in formamide (Sigma-Aldrich) for 48 h at 37 °C to extract the dye. Absorbance was measured at 620 nm. The concentration of Evans blue in the brain was calculated using a standard curve prepared with known concentrations of Evans blue in formamide.

### Flow cytometric analysis of the brain

Brains were digested in RPMI containing 1.6 mg/mL collagenase (type IV; Sigma-Aldrich) and 200 µg/mL DNase I (Sigma-Aldrich) at 37 °C for 50 min. Cells were isolated using a Percoll gradient (GE Healthcare) and debris was filtered out using a 70 µm nylon mesh. Cells were counted and labelled with LIVE/DEAD amine-reactive violet viability marker according to the manufacturer’s protocol (Invitrogen). Cells were labeled with FITC anti-CD45 (eBioscience; 30-F11), PE anti-CD11b (BD Pharmingen; M1/70), PerCP-Cy5.5 anti-CD4 (eBioscience; RM4–5) and APC-eFluor780 anti-CD8 (eBioscience; 53–6.7). Flow cytometry was performed using a BD LSR Fortessa and results were analyzed using FlowJo version 9.6.2. Mice were perfused for the analysis of brain sequestered cells.

### Flow cytometric analysis of the spleen

Splenocytes were isolated and erythrocytes were lysed in Tris-NH_4_Cl buffer. To analyze cytokine production by T cells, isolated splenocytes were stimulated with 50ng/mL phorbol 12-myristate 13-acetate (PMA) and 500ng/mL ionomycin for 5 h at 37 °C. Brefeldin A (eBioscience) was added for the entire duration of the experiment. For the analysis of cytokine production by B cells, isolated splenocytes were stimulated with 50ng/mL PMA, 500ng/mL ionomycin and 10 µg/mL LPS (Sigma) for 5 h at 37 °C. Monensin (eBioscience) was added for the entire duration of the experiment. *Ex vivo* stimulation was performed in IMDM supplemented with 10% FBS (Invitrogen), 100 U/mL penicillin, 100 μg/mL streptomycin and 2 mM L-glutamine (Wisent), and 50 μM 2β-mercaptoethanol (Sigma-Aldrich). Cells were counted and labelled with PerCP-Cy5.5 anti-CD4 (eBioscience; RM4–5), APC-eFluor780 anti-CD8 (eBioscience; 53–6.7), APC anti-CXCR3 (eBioscience; CXCR3–173), FITC anti-CD62L (eBioscience; MEL-14), PE-Cy7 anti-CD44 (eBioscience; IM7), eFluor 450 anti-CD11a (eBioscience; M17/4), FITC anti-IL-10 (BD Pharmingen, JES5-16E3), BV785 anti-IFNγ (BioLegend; XMG1.2), PE anti-CD223 (LAG-3) (eBioscience; eBioC9B7W), APC anti-Foxp3 (eBioscience; FJK-16s), eFluor 450 anti-CD49b (integrin α2; DX5), and PE anti-CD19 (eBioscience; eBio1D3). Flow cytometry was performed using a BD LSR Fortessa and a BD FACSCanto II and results were analyzed using FlowJo version 9.6.2.

### *Ex vivo* induction of CXCR3 on splenic T cells

CD4^+^ and CD8^+^ T cells were isolated from total splenocytes using the EasySep^TM^ Mouse CD4^+^ T Cell Isolation Kit and the EasySep^TM^ Mouse CD8^+^ T cell Isolation Kit, respectively, according to the manufacturer’s protocol (Stemcell). 5 μg/mL anti-CD3 (eBioscience; 145-2C11) and 2 μg/mL anti-CD28 (eBioscience; 37.51) were co-immobilized on 24-well plates overnight at 4 C° and wells were washed 3 times with PBS prior to T cell stimulation. 1.5 × 10^6^ CD4^+^ or CD8^+^ T cells were incubated at 37 °C for 48 h in RPMI supplemented with 10% FBS (Invitrogen/Wisent), 100 U/mL penicillin, 100 μg/mL streptomycin and 2 mM L-glutamine (Wisent/Sigma), with or without 1 μM bpV(phen). Cells were collected and incubated for an additional 24 h in the absence of anti-CD3 and CD28 in RPMI with or without 1 μM bpV(phen). Cells were counted and labelled with PerCP-Cy5.5 anti-CD4 (eBioscience; RM4-5), APC-eFluor780 anti-CD8 (eBioscience; 53–6.7), and APC anti-CXCR3 (eBioscience; CXCR3-173). Flow cytometry was performed using a BD LSR Fortessa and results were analyzed using FlowJo version 9.6.2.

### CD3 cross-linking of splenic CD8 T cells

Splenic CD8^+^ T cells were isolated as described above and 2.0–10.0 × 10^6^ CD8^+^ T cells were stimulated on 6-well plates using the same protocol that was utilized for the *ex vivo* induction of CXCR3 on splenic T cells. 0.5–10.0 × 10^6^ cells were incubated on ice with 10 μg/mL biotinylated CD3 (eBioscience; 145–2C11) and cross-linked with streptavidin for the indicated times at 37 °C. Cells were washed with ice cold PBS and lysed in NP40 buffer (20 mM HEPES, pH 7.9; 100 mM NaCl; 5 mM EDTA; 0.5 mM CaCl; 1% Nonidet p-40; 1 mM PMSF; 10 μg/mL leupeptin; 5 μg/mL pepstatin; and 1 mM Na_3_VO_4_). 5–15 μg of protein was resolved by SDS-PAGE, transferred to a methanol-activated PVDF membrane, and probed with antibodies as indicated. Antibodies were detected with goat anti-rabbit conjugated to horseradish peroxidase (Santa Cruz; sc-2054) and HyGlo (Denville Scientific). Images were quantified with ImageJ software. Phosphorylation quantification is presented as the ratio of signal intensity of the phosphorylated protein of interest to the signal intensity of total protein, and was normalized to the maximal phosphorylation.

### Quantification of alanine and aspartate transferase

Blood samples were collected by cardiac puncture and the serum samples were analyzed by the McGill University Comparative Medicine and Animal Resources Centre.

### Quantification of liver parasite burden

Pieces of liver were homogenized in PBS containing 0.1 mg/mL aprotinin (Roche), 0.05 mg/mL leupeptin (Roche) and 1X Firefly lysis buffer using a PRO200 Hand-held Homogenizer (Harvard Apparatus Canada), and lysed on ice. The homogenate was centrifuged at 13,000 rpm for 30 min and the luciferase activity of the supernatant was measured using the Firefly Luciferase Assay Kit (Biotium, Inc.), according to the manufacturer’s protocol.

### Statistical analysis

Statistical analyses were performed using the unpaired Student’s *t*-test (two-tailed) or one-way ANOVA and Tukey’s multiple comparisons test. Error bars represent S.E.M. The log-rank test was used for all experiments in which survival was assessed as an endpoint. The data were analyzed using GraphPad Prism software.

## Electronic supplementary material

Supplementary Information
