# Supplementary material for: Protein Tyrosine Phosphatase Inhibition Prevents Experimental Cerebral Malaria by Precluding CXCR3 Expression on T Cells
Source: Sci Rep. 2017 Jul 14;7:5478. doi: 10.1038/s41598-017-05609-1 (PMC5511231; doi:10.1038/s41598-017-05609-1)
Supplement: Supplementary file 1 — Supplementary Information [file 41598_2017_5609_MOESM1_ESM.pdf]

Protein Tyrosine Phosphatase Inhibition Prevents Experimental Cerebral Malaria by Precluding CXCR3 Expression on T Cells

Kristin M. Van Den Ham[1,3], Logan K. Smith[1,2], Martin J. Richer[1,2*] and Martin Olivier[1,3*]

[1]Department of Microbiology and Immunology, McGill University, Montréal, QC, H3A 0G4, Canada, [2]Microbiome and Disease Tolerance Centre and Associate Member, Goodman Cancer Research Centre, McGill University, Montréal, QC, H3A 2B4, Canada, [3]Infectious Diseases and Immunity in Global Health Program, Research Institute of the McGill University Health Centre, Montréal, QC, H4A 3J1, Canada.

*Corresponding authors: martin.olivier@mcgill.ca, martin.j.richer@mcgill.ca

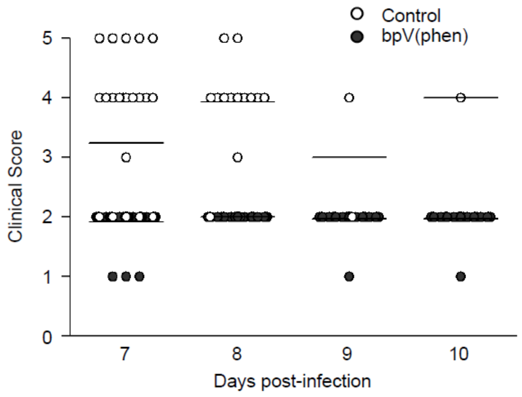

Supplementary Figure S1. PTP inhibition prevents the development of neurological symptoms. ECM clinical score. 0 = healthy mouse; 1 = ruffled fur; 2 = panting; 3 = hunching, wobbly gait; 4 = paralysis, convulsions; and 5 = coma. The cumulative average of 5 independent experiments is shown for clinical score. *n* = 29 for control mice and *n* = 34 for bpV(phen)-treated mice for clinical score.

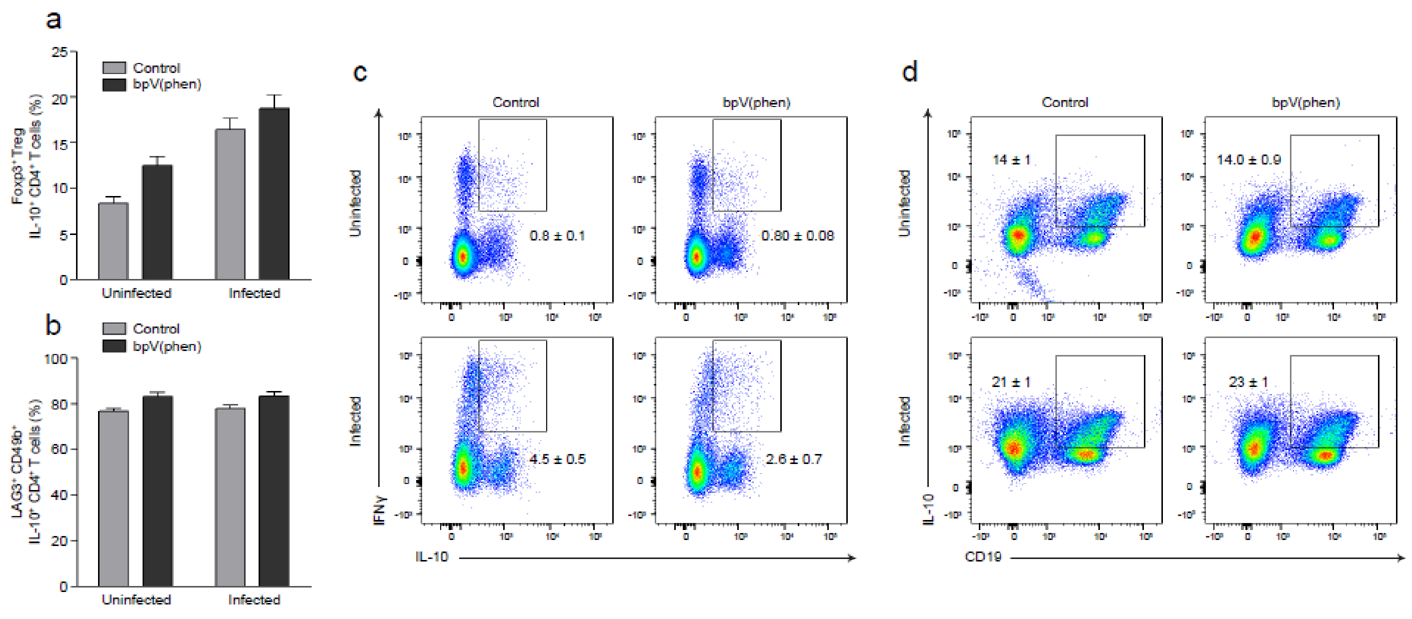

Supplementary Figure S2. IL-10 production by CD4$^+$ T cells and B cells. Percentage of IL-10$^+$ CD4$^+$ T cells that (**A**) Foxp3$^+$ and (**B**) LAG3$^+$CD49$^+$, and representative flow cytometry plots of (**C**) IFNγ$^+$IL-10$^+$ CD4$^+$ T cells measured on day 7 post-infection after PMA/ionomycin stimulation. Representative flow cytometry plots of (**D**) IL-10$^+$ B cells measured on day 7 post-infection after PMA/ionomycin/LPS stimulation. The numbers shown on the flow cytometry plots indicate the mean percentage of cells inside the gate ± S.E.M. For the graphs, the cumulative average of 2 independent experiments is shown; *n* = 9 for uninfected, control mice, *n* = 9 for uninfected, bpV(phen)-treated mice, *n* = 11 for infected, control mice, and *n* = 11 for infected, bpV(phen)-treated mice. For the IFNγ$^+$IL-10$^+$ CD4$^+$ T cells, *n* = 5 for uninfected, control mice, *n* = 4 for uninfected, bpV(phen)-treated mice, *n* = 5 for infected, control mice, and *n* = 4 for infected, bpV(phen)-treated mice. For the IL-10$^+$CD19$^+$ cells, *n* = 5 for all groups.

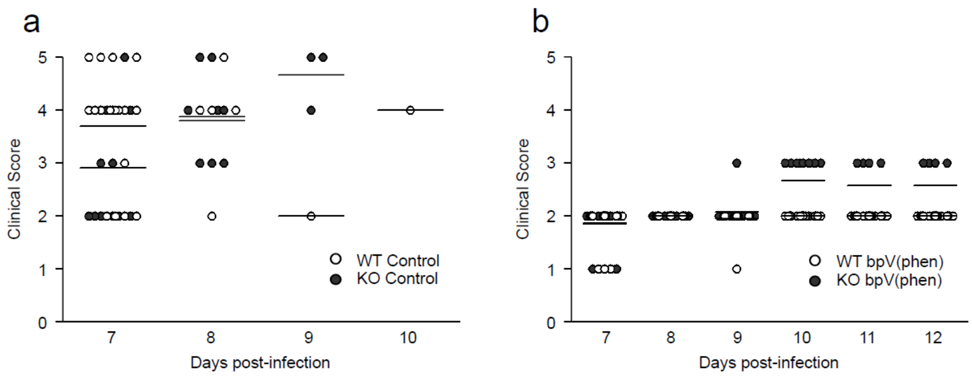

Supplementary Figure S3. PTP inhibition prevents the development of neuropathology in IL-10KO mice. ECM clinical score of (**A**) control WT and IL-10KO mice and (**B**) bpV(phen)-treated WT and IL-10KO. 0 = healthy mouse; 1 = ruffled fur; 2 = panting; 3 = hunching, wobbly gait; 4 = paralysis, convulsions; and 5 = coma. The cumulative average of 4 independent experiments is shown for clinical score. n = 17 for control, WT mice, n = 22 for bpV(phen)-treated, WT mice, n = 12 for control, IL-10KO mice and n = 13 for bpV(phen)-treated, IL-10KO mice.

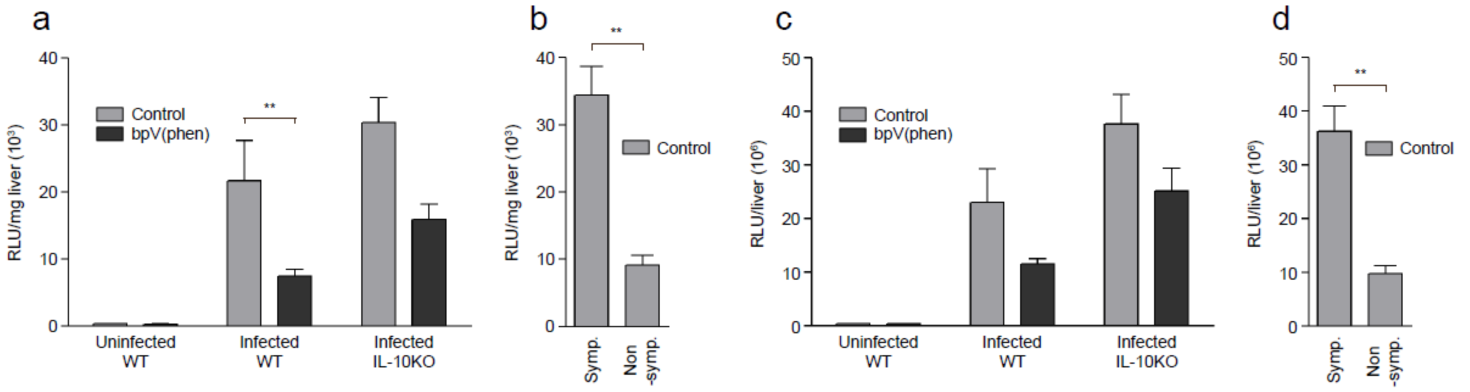

Supplementary Figure S4. Liver parasite load is dependent on the onset of cerebral symptoms. (**A**) RLU per milligram of liver of WT and IL-10KO mice and (**B**) RLU per milligram of liver of symptomatic and non-symptomatic control, WT mice. (**C**) Total liver RLU of WT and IL-10KO mice and (**D**) total liver RLU of symptomatic and non-symptomatic control, WT mice. Liver parasite burden was measured on day 7 (control WT and IL-10KO mice) and day 10 (bpV(phen)-treated WT and IL-10KO mice) post-infection. ** $P < 0.01$ using a one-way ANOVA and Tukey's multiple comparisons test (A) and using the unpaired t-test (B and D). n = 5 for control, WT mice, n = 8 for bpV(phen)-treated, WT mice, n = 4 for control, IL-10KO mice and n = 4 for bpV(phen)-treated, IL-10KO mice.

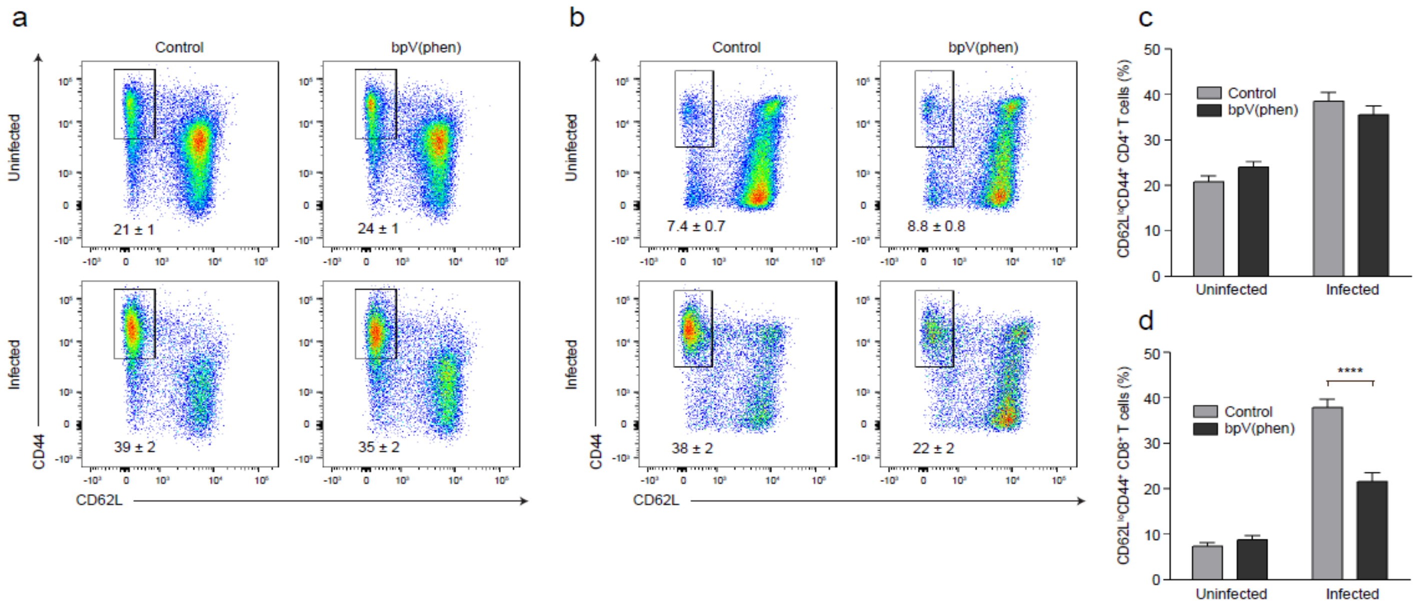

Supplementary Figure S5. PTP inhibition decreases the activation of splenic CD8$^+$ T cells. Representative flow cytometry plots of CD62L^{lo}CD44$^+$ (**A**) CD4$^+$ T cells and (**B**) CD8$^+$ T cells, and the percentage of CD62L^{lo}CD44$^+$ (**C**) CD4$^+$ T cells and (**D**) CD8$^+$ T cells measured on day 7 post-infection. The numbers shown on the flow cytometry plots indicate the mean percentage of cells inside the gate ± S.E.M. The cumulative average of 2 independent experiments is shown. n = 9 for uninfected, control mice, n = 10 for uninfected, bpV(phen)-treated mice, n = 9 for infected, control mice, and n = 9 for infected, bpV(phen)-treated mice. **** P < 0.0001 for the percentage of CD62L^{lo}CD44$^+$ CD8$^+$ T cells using a one-way ANOVA and Tukey's multiple comparisons test.

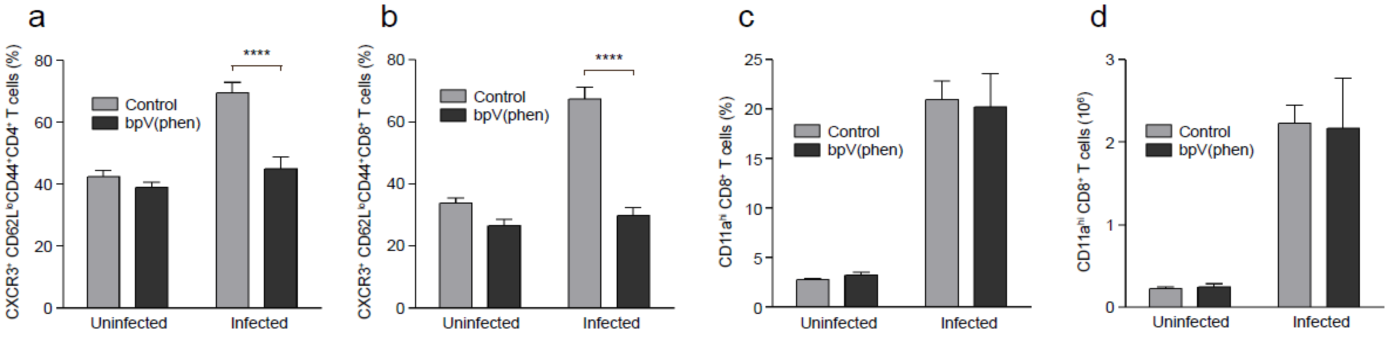

Supplementary Figure S6. bpV(phen) decreases CXCR3 on activated T cells, but does not affect the antigen experience of CD8$^+$ T cells. The percentage of CXCR3$^+$ CD62L^{lo}CD44$^+$ (**A**) CD4$^+$ T cells and (**B**) CD8$^+$ T cells, and the (**C**) percentage and (**D**) total number of antigen-experienced (CD11a^{hi}CD8^{lo}) CD8$^+$ T cells measured on day 7 post-infection. The cumulative average of 2 independent experiments is shown; n = 9 for uninfected, control mice, n = 10 for uninfected, bpV(phen)-treated mice, n = 9 for infected, control mice, and n = 9 for infected, bpV(phen)-treated mice (A and B). n = 6 for uninfected, control mice, n = 6 for uninfected, bpV(phen)-treated mice, n = 11 for infected, control mice, and n = 7 for infected, bpV(phen)-treated mice (C and D). **** P < 0.0001 using a one-way ANOVA and Tukey’s multiple comparisons test.

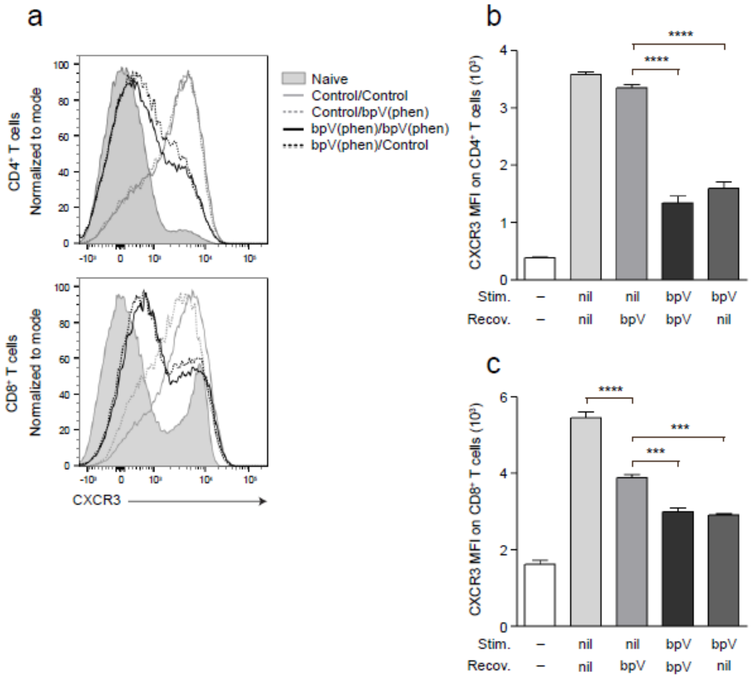

Supplementary Figure 7. PTP inhibition attenuates CXCR3 expression to a greater extent during stimulation compared to recovery. (**A**) Representative histograms of CXCR3 expression on bpV(phen)-treated CD4$^+$ and CD8$^+$ T cells following anti-CD3/anti-CD28 stimulation, and the mean fluorescence intensity (MFI) of CXCR3 on bpV(phen)-treated (**B**) CD4$^+$ and (**C**) CD8$^+$ T cells following stimulation. The cumulative average of 3 independent experiments is shown. *** $P < 0.001$ and **** $P < 0.0001$ using a one-way ANOVA and Tukey's multiple comparisons test.
